# “I am scared, I do not want to lie”: exploring the impacts of COVID-19 on engagement in care, perceived health, relationship dynamics, and parenting among postpartum women with HIV in South Africa

**DOI:** 10.1186/s12884-023-05520-w

**Published:** 2023-04-03

**Authors:** Amelia M. Stanton, Abigail P. Blyler, Nzwakie Mosery, Georgia R. Goodman, Rachel Vanderkruik, Kedibone Sithole, C. Andres Bedoya, Jennifer Smit, Christina Psaros

**Affiliations:** 1 Department of Psychological and Brain Sciences, Boston University, Boston, MA USA; 2 Department of Psychiatry, Massachusetts General Hospital, Boston, MA USA; 3 The Fenway Institute, Fenway Health, Boston, MA USA; 4 Department of Psychology, Positive Psychology Center, University of Pennsylvania, Pennsylvania, PA USA; 5 Wits MatCH Research Unit (WMRU), Department of Obstetrics and Gynaecology, Faculty of Health Sciences, University of the Witwatersrand, Johannesburg, South Africa; 6 Department of Emergency Medicine, Brigham and Women’s Hospital, Boston, MA USA; 7 Harvard Medical School, Boston, MA USA

**Keywords:** HIV, Women, COVID-19, Postpartum, Engagement in care

## Abstract

**Background:**

COVID-19 and efforts to manage widespread infection may compromise HIV care engagement. The COVID-19-related factors linked to reduced HIV engagement have not been assessed among postpartum women with HIV, who are at heightened risk of attrition under non-pandemic circumstances. To mitigate the effects of the pandemic on care engagement and to prepare for future public health crises, it is critical to understand how COVID-19 has impacted (1) engagement in care and (2) factors that may act as barriers to care engagement.

**Methods:**

A quantitative assessment of COVID-19-related experiences was added to a longitudinal cohort study assessing predictors of postpartum attrition from HIV care among women in South Africa. Participants (*N* = 266) completed the assessment at 6, 12, 18, or 24 months postpartum between June and November of 2020. Those who endorsed one or more challenge related to engagement in care (making or keeping HIV care appointments, procuring HIV medications, procuring contraception, and/or accessing immunization services for infants; *n* = 55) were invited to complete a brief qualitative interview, which explored the specific factors driving these challenges, as well as other impacts of COVID-19 on care engagement. Within this subset, 53 participants completed an interview; qualitative data were analyzed via rapid analysis.

**Results:**

Participants described key challenges that reduced their engagement in HIV care and identified four other domains of COVID-19-related impacts: physical health, mental health, relationship with a partner or with the father of the baby, and motherhood/caring for the new baby. Within these domains, specific themes and subthemes emerged, with some positive impacts of COVID-19 also reported (e.g., increased quality time, improved communication with partner, HIV disclosure). Coping strategies for COVID-19-related challenges (e.g., acceptance, spirituality, distraction) were also discussed.

**Conclusions:**

About one in five participants reported challenges accessing HIV care, medications, or services, and they faced complex, multilayered barriers to remaining engaged. Physical health, mental health, relationships with partners, and ability to care for their infant were also affected. Given the dynamic nature of the pandemic and general uncertainty about its course, ongoing assessment of pandemic-related challenges among postpartum women is needed to avoid HIV care disruptions and to support wellbeing.

## Introduction

As of January 2023, over 4 million COVID-19 infections and over 100,000 COVID-19-related deaths have been reported in South Africa (SA), figures which likely underestimate the spread of the virus and associated mortality [1]. Current estimates indicate that just over 32% of the SA population has completed the initial vaccination protocol, leaving the majority of individuals in SA unprotected from severe disease [1]. Of relevance to public health in this setting, specific populations are at higher risk for severe morbidity from COVID-19, including individuals with HIV [2, 3] and adults with compromised immune systems [4]. Increasing the complexity of COVID-19 risk management, SA is home to the largest HIV epidemic in the world, with 7.8 million adults and children living with HIV [5]. HIV prevalence rates are higher among women aged 45 and younger (24.7%) compared to men (13.5%) [5], indicating that women of reproductive age bear the greatest HIV burden and are therefore at high risk for detrimental COVID-19-related outcomes.

In the context of COVID-19 and repeated periods of lockdown implemented by the SA government, there was and continues to be a strong possibility that people with HIV (PWH) might lose access to their HIV care (even for a temporary period), with particularly negative implications for postpartum women. To optimize maternal health and decrease perinatal transmission, engagement in HIV care is critical during the postpartum period. In what is commonly referred to as the prevention of mother-to-child transmission (PMTCT) cascade, women must complete a series of steps (e.g., administer antiretroviral therapy (ART) to the infant, ensure that infants are tested for HIV, adhere to breastfeeding recommendations of six to 24 months) [6] to reduce the likelihood of HIV transmission during pregnancy, labor, and breastfeeding. With PMTCT interventions, the average rate of perinatal transmission is approximately 3.5% [7]; to achieve this rate, women must effectively navigate adherence barriers to avoid falling off the treatment cascade “cliff.” [8].

Maintaining engagement in HIV care during the postpartum period can be difficult under the best of circumstances. Some of the challenges to remaining in care that are unrelated to COVID-19 include stigma [9] (i.e., from providers as well as internalized stigma and shame associated with HIV), structural barriers [10, 11] (e.g., the need to transfer to a new clinic after delivery), and decreased motivation to remain adherent to ART after delivering an HIV-negative infant [12]. In addition to remaining engaged in HIV/PMTCT care, postpartum women with HIV (WWH) also need to manage infant care appointments, including immunization visits, and adjust to the stressors of new parenthood and/or the complexities of caring for other children while also meeting the needs of a newborn. Then, during multiple COVID-19-related national lockdowns, postpartum WWH had to balance the need to visit clinics to obtain care for themselves and their infants, facing all of the challenges described above, with additional risk of (a) the spread of a highly infectious, possibly fatal respiratory disease, and (b) limited access to usual medical care if lockdown regulations were not followed [13].

In addition to these unique engagement in care challenges, the mental health and relational stressors already faced by perinatal WWH in SA may have been exacerbated by the COVID-19 pandemic. In sub-Saharan countries with high HIV prevalence rates, perinatal WWH have elevated rates of depression [14] (with rates approaching 50% in SA) [15], reduced social support [16], and fears about the physical wellbeing of their children [17–19] given the possibility of HIV transmission. Moreover, both pregnant and nonpregnant WWH experience high rates of intimate partner violence (IPV) and sexual trauma, contributing to rates of depression and posttraumatic stress disorder [20]. Such experiences may lead to the development of avoidance-based coping strategies [21, 22], which, together with COVID-19 restrictions, may have further compromised engagement in care and limited opportunities to seek social support from individuals outside of their households. In addition, among WWH experiencing IPV prior to the emergence of COVID-19, lockdown periods may have negatively impacted their safety and the safety of their children. Notably, for perinatal WWH who had not disclosed their HIV status to their abusive partners, accessing HIV care and PMTCT services could place them at increased risk for violence [13]. Economic stressors that are common among perinatal WWH during non-pandemic conditions— unemployment or underemployment, inability to buy food for themselves and their infants, limited funds for transportation to the clinic—may have also intensified during the most restrictive levels of the SA government’s COVID response, further compromising mental health and potentially exacerbating relationship difficulties. Among pregnant and postpartum girls and adolescents in Uganda and SA, unmet financial and material expectations, particularly from pregnancy partners, have been reported to be major sources of stress [23, 24]. A lack of financial independence from partners may compromise mental health via increased risk for IPV [25] and serve as a barrier to seeking and accessing mental health care, which could have downstream effects on HIV care engagement.

With the COVID-19 pandemic now in its third year, long-term management of risk and negative health outcomes will be critical in all contexts but particularly complex in resource limited settings like SA. Here, specific subpopulations, including postpartum WWH, will likely continue to experience COVID-19-related physical and psychological health disparities for decades to come. To actively mitigate these disparities, we must explore the multifaceted impacts of the pandemic on engagement in HIV- and infant-related care, as well as on the domains that influence care engagement, including mental health, intimate relationships or partnerships, and associated coping strategies. In this qualitative sub-study, we leveraged an existing cohort to speak directly to postpartum WWH about these intersecting challenges, with the ultimate goal of reducing care disruptions over the course of the current pandemic and during future public health crises.

## Methods

### Parent study procedures

The parent trial was a prospective longitudinal cohort study based in Durban, KwaZulu-Natal, SA that had two main goals: (1) to estimate the rate of attrition from HIV care during the postpartum period and (2) to identify factors associated with attrition. Data collection was initiated in February 2018 and completed in June 2022, and the primary data analyses are currently underway.

The aim and procedures of the parent study are described in detail elsewhere [26]. Briefly, the primary quantitative outcomes of the study are HIV RNA (i.e., viral suppression) and self-reported number of visits to any HIV care provider (i.e., retention in care). Four-hundred seventy-two women with HIV between the ages of 18 and 45 were recruited at 28 weeks of pregnancy or greater. Other inclusion criteria included (1) currently taking antiretroviral therapy, (2) fluent in English or isiZulu, (3) access to a phone and willing to be contacted for repeated assessments, and (4) ability to provide informed consent. Women were excluded from the parent study if they had an active or untreated major mental illness that would interfere with participation (e.g., untreated psychosis, bipolar disorder, active suicidality) or if participation would compromise their safety (i.e., would put them at risk for violence). The study included five assessment timepoints: at baseline (during pregnancy) and follow-up at six, 12, 18, and 24 months post-baseline.

### Sub-study procedures

A brief quantitative assessment and a qualitative interview exploring COVID-19-related challenges to engagement in care and other associated concerns were added to the assessment battery of the parent study at all follow-up time points (six, 12, 18, and 24 months). At the end of follow-up assessments conducted between 1 June and 30 November 2020 (all telephonic), study staff asked all participants if they were willing to answer some additional questions about their experiences during the ongoing COVID-19 pandemic. Participants who agreed to do so remained on the phone to complete both the quantitative assessment and the brief qualitative interview. For the present study, we analyzed only the qualitative interviews of participants who reported at least one engagement in care challenge in the quantitative assessment.

Importantly, SA follows a five-level COVID-19 alert system based on epidemiological trends, health system capacity to respond to disease burden, and any other factors that might influence rates of infection, hospitalization, and mortality. The system ranges from level 1 (“low COVID-19 spread with high health system readiness”) to level 5 (“high COVID-19 spread with high health system readiness”) [27]. From June to November 2020, alert levels ranged from level 1 to level 3 (level 3 from June 1 to August 17, level 2 from August 18 to September 20, and level 1 from September 21 through the end of November). Notably, the country went into full lockdown (level 5) from March 26 to April 30, 2020, which was then decreased to level 4 on May 1.

Data for the sub-study were collected across two COVID-19 waves, defined by the SA government as the period from which COVID-19 weekly incidence is equal to or greater than 30 cases per 100,000 persons until the weekly incidence is equal or below 30 cases per 100,000 persons [27]. Therefore, the first wave lasted from week 24 of 2020 (June 8–14) to week 34 of 2020 (August 17–23), and the second wave from week 47 of 2020 (November 16–22) to week five of 2021 (February 1–7).

### Quantitative assessment

We utilized a brief quantitative assessment that included items adapted from the N2 COVID-19 Check-in Survey Items, as well as from the Adolescent Trials Network COVID Questionnaire Draft [28], and questions generated by our team. Proposed items were drafted by several US- and SA-based team members (AMS, NM, KS, CAB, JS, CP), translated and pilot tested in the field with 1–2 participants by SA-based team members (NM, KS), then refined by the larger team if language was unclear. The assessment was used to identify women who faced challenges (1) making or keeping their HIV care appointments (“Have you had trouble making or keeping your HIV care appointments with your health care provider during the COVID-19 pandemic?”), (2) procuring their HIV medications (“Have you had trouble getting your HIV medication from your health care provider during the COVID-19 pandemic?”), (3) procuring contraception (“Have you had trouble getting your contraceptive method during the COVID-19 pandemic?”, “Have you had trouble accessing condoms during the COVID-19 pandemic?”), and (4) accessing immunization services for their infants (“Have you had trouble accessing immunization services for your baby during the COVID-19 pandemic?”). Participants were provided dichotomous (yes/no) options to the four questions above; if “yes” was endorsed, participants were then prompted to select from a list of options to identify the specific factors that contributed to these challenges (e.g., for the first question, sample options included limited transportation, limited appointment availability, limited availability of services, lack of childcare). To characterize the sample with respect to COVID-19-related care, participants were also asked about COVID-19 diagnoses and treatment (e.g., whether they had been screened/tested for COVID-19, received a COVID-19 diagnosis, received associated treatment).

In some cases, participants completed the quantitative assessment more than once (i.e., at more than one follow-up assessment); on these occasions, their first assessment was used to identify whether or not their qualitative data would be included in this analysis.

### Qualitative assessment

The qualitative assessment explored factors associated with the challenges identified in the quantitative survey, as well as participants’ perceptions of the impact of the pandemic on perceived health risks, mental health, intimate partnerships, and ability to care for their infants. Individual qualitative interviews were conducted telephonically using a semi-structured interview guide that followed best practice procedures established by Huberman and Miles [29] and Strauss and Corbin [30]. Some items were adapted from the Bennett and Elliott Qualitative Interview Guide, as well as from the Gwadz Qualitative Interview Guide, both of which were accessed in April 2020 via an open-sourced document developed by the Center for Drug Use and HIV Research [28] in the early stages of the COVID-19 pandemic. Items probing aspects of partnerships and experiences as a new mother were added. The broad content areas of the qualitative interview, as well as sample questions and problems, are provided in Table 1.

**Table 1:** Sample interview content areas, questions, and probes

Content area: *Impact of COVID-19 on…*	Sample questions and probes
Engagement in HIV care and overall health	• Which are the biggest risks to your health right now? Why do you feel that way? • What would help you the most right now in terms of limiting those risks or feeling safer? • What stands in the way of that?
Mental health and wellbeing	• How has the coronavirus outbreak impacted your mental health and wellbeing? •How has the COVID-19 pandemic affected your mood?
Relationship with partner	• Have you experienced any physical violence from your partner during the COVID-19 pandemic? • Have you been separated from your partner during the COVID-19 pandemic? If yes, how did the separation affect your relationship? • Please described any positive experiences that you have had with your partner during the COVID-19 pandemic
Experiences as a new mother	• How has the COVID-19 pandemic impacted your financial support? Social support? • How has the COVID-19 pandemic impacted your family’s ability to help you with the baby? • How do these experiences or feelings affect your ability to manage your health?
Coping	• What has helped you the most to cope during the COVID-19 pandemic? • What has helped you the most with…taking your ARVs, caring for your child(ren), demand for food and other needs, your family and partner?

### Analyses

Overall, 266 postpartum WWH completed the COVID-19 quantitative assessment across all follow-up time points; 34.0% completed the assessment at the six-month follow-up visit, 24.5% completed the questions at the 12-month visit, 32.1% at the 18-month visit, and 9.4% at the 24-month visit. Of the 266 participants who completed the COVID-19 quantitative assessment, 55 (20.7%) endorsed at least one COVID-19-related engagement in care issue. Among this subset, challenges making and keeping HIV care appointments were reported by 8.2% (*n* = 22) of participants, 4.9% (*n* = 13) reported difficulties accessing HIV medications, 7.5% (*n* = 20) noted problems securing contraception, and 5.2% (*n* = 14) indicated challenges accessing infant immunization services. Fifty-three of the 55 participants (96.4%) who quantitatively reported challenges agreed to complete a brief, targeted qualitative interview, and descriptive statistics were calculated only among these 53 participants.

All interviews were digitally recorded, transcribed, and translated from isiZulu to English by SA team members (NM, KS) who identify as Black South Africans and have both expertise and lived experiences with the isiZulu culture. To facilitate quick dissemination of study findings locally among stakeholders in Durban, we used a rapid analysis approach [31]. Following this approach, four study team members (AMS, APB, GRG, RV) trained in qualitative methods independently reviewed the same subset of transcripts using a template of neutral domain names, which were selected to correspond with each interview question (e.g., mental health, motherhood, coping; see Table 1). The reviewers reduced and summarized the data using the template, then organized the summaries into themes and subthemes. All four team members met on an ongoing basis to compare the text that they had organized under each domain, their associated themes, and sample text that they had selected to highlight those themes for the subset of interviews that they all coded. During these meetings, they resolved any domain, theme, and subtheme discrepancies; then, they were each assigned a batch of interviews to code independently. After all interviewers were coded, the team met one additional time to collate the data, discuss all themes and subthemes, reach consensus on final themes, and review extracted example quotes to present in the tables.

## Results

### Characteristics of sample

The average age of the sample (*n* = 53) was 29.4 years (SD = 5.3), and 58.5% (*n* = 31) of participants were diagnosed with HIV more than five years ago. Almost all participants identified as Black South African (98.1%, *n* = 52). Most (77.4%, *n* = 41) had completed up to Standard 10 at school (the equivalent of 11^th^ or 12^th^ grade in secondary school), 77.4% (*n* = 41) were unemployed, and 90.6% (*n* = 48) had a monthly income of less than 4000 South African Rand (ZAR) or roughly $330 USD. Approximately 17.0% (*n* = 9) of the sample reported living with a romantic partner, and 86.8% (*n* = 46) of participants had a long-term partner but were not married. The average number of living children across the sample was 1.7 (range: 1–4). Full sociodemographic characteristics are provided in Table 2. To explore factors that may be driving engagement in care challenges, we assessed for differences in certain demographic factors (age, number of children, employment, years since HIV diagnosis) between the larger sample (*N* = 266) and the sub-sample (*n* = 53). No significant differences emerged.

**Table 2:** Sociodemographic and select characteristics of sample (*N* = 53)

	*n*	%
Sociodemographics		
Age (in years)		
Mean (SD)	29.4 (5.3)	–
Range	19–41	–
Race		
Black South African	52	98.1
Mixed or multiracial	1	1.9
Education		
Standard 6 (8^th^ grade)	2	3.8
Standard 7 (9^th^ grade)	3	5.7
Standard 8 (10^th^ grade)	5	9.4
Standard 9 (11^th^ grade)	12	22.6
Standard 10 (12^th^ grade)	19	35.8
Some post-secondary (University, College, Vocational)	8	15.1
Completed post-secondary training	4	7.5
Employment		
Full-time	7	13.2
Part-time	5	9.4
Unemployed	41	77.4
Maternity leave offered (among those employed full- or part-time)	6	50.0
Monthly income in ZAR (current USD value)		
0 to 499 ($0 to $33.14)	4	7.5
500 to 999 ($33.20 to $66.34)	13	24.5
1000 to 1999 ($66.41 to $132.75)	18	33.9
2000 to 2999 ($132.82 to $199.16)	7	13.2
3000 to 3999 ($199.22 to $265.56)	6	11.3
4000 to 4999 ($265.63 to $331.97)	4	7.5
Over 5000 (Over $332.04)	1	1.9
Sources of income (option to select > 1)		
Employment	12	11.8
Self-generated income	10	9.8
Government grant	30	29.4
Money from partner(s)	35	34.3
Money from family	15	14.7
Living with^a^		
Self	9	17.3
Partner/spouse	9	17.3
Family members	26	50.0
Roommates	2	3.8
Children	10	19.2
Number of living children		
Mean (SD)	1.7 (0.9)	–
Range	1–4	–
Relationship with father of current pregnancy		
Married	1	1.9
Engaged to be married	3	5.7
Long-term partner (main partner for ≥ 1 year OR living together, but not married or engaged)	46	86.8
Boyfriend(s) (main partner for < 1 year, not living together)	3	5.7
Years since HIV diagnosis		
Five years or less	22	41.5
Five years or more	31	58.5
**COVID-19-related**^b^	***n***	**%**
Screened/tested for COVID-19	47	95.9
Diagnosed with COVID-19	1	2.0
Received treatment for COVID-19	1	2.0
**Interview timepoint**	***n***	**%**
Follow-up visit		
6 months	18	34.0
12 months	13	24.5
18 months	17	32.1
24 months	5	9.4
Corresponding COVID-19 wave [27]		
Wave 1 (approx. June 2020 – August 2020)	48	90.6
Wave 2 (approx. November 2020 – February 2021)	5	9.4

^a^Data missing from one participant, so *n* = 52 for this item. Participants had the option to select multiple types of cohabitants

^b^Data missing from four participants, so *n* = 49 for this item

### Qualitative findings

The qualitative interviews yielded numerous themes and subthemes within five key domains: engagement in HIV care, physical health, mental health, relationship with partner/father of the baby, and motherhood and caring for the new baby. See Table 3 for sample extracts from the interviews, categorized by domain, theme, and subtheme.

**Table 3:** Impacts of COVID-19 across domains

Domains, associated themes, and subthemes	Illustrative quotes
**ENGAGEMENT IN HIV CARE**	
***Impacts on ART use:***	
Missed ART doses	*“Ja It got finished and I stayed for two weeks without. I kept going to clinic thinking maybe if I try my luck and go maybe I will find someone who will understand… But they all refused, and I went to the sick people thinking that I will meet someone, but it is still the same. They refused.” (Age 23)*
Used others’ extra ART pills	*“It does not happen, I make sure that I collect them even if they get finished, many of us take it, so there is even extra, you see. I have never missed my treatment ever since the lock down started… There are three people who are taking pills. They have extra pills, you see, which they have not used, you see. They give me from their pills.” (Age 22)*
Challenges picking up ART due to:	
Clinic closures	*“It has affected me in that sometimes the clinics close, when you go to the clinic to…collect your pills and you would find that they are not working, maybe they found someone who is sick, who has COVID-19. I have been to [name of clinic] and they were closed, and I waited for a couple of days and then I heard from others that they are open, and I went back and got my pills.” (Age 21)*
Limits on timing of public transportation	*“So, we were really disturbed at that time, you will find that you take transport to collect your pills you stand there until it is time for you to go back and then you rush back again because at 10:00 the transport finishes and then the transport operates again at 16:00 and that is when they close.” (Age 37)*
Lack of money for transportation	*“It is my life, but I may end up not getting them accordingly because when I send someone from that side, I do not have money, I am not working. I do not have transport money to go there, do you see that.” (Age 30)*
Lack of documentation to provide to clinic	*“It was difficult because I was in the village and they did not give us…they did not want to understand that we need to get to the clinic. I really struggled… They cannot just give you medication when they do not have anything in writing on what pills you are taking. but I explain that we did not know that this lockdown was going to happen.” (Age 23)*
**PHYSICAL HEALTH**	
***Contracted COVID-19***	*“I went to the hospital to get tested and they called me and told me that I was positive, so they told me to isolate. I stayed until I showed symptoms but when I tested I did not have any symptoms…What happened was I had no sense of smell and taste after I had received my results and then started coughing which came with shortness of breath and my mom said I should go to the hospital for oxygen and then it was okay.” (Age 29)*
*** High risk for COVID-19 due to:***	
HIV status	*“I would say…my risk as a person who is positive, I am at a high risk if I can get this virus because I am not hundred percent sure if I will survive… It is only God knows but as a person who is HIV positive, I cannot treat myself as if I am alright. I am not scared of anything—no I am scared, I do not want to lie I am scared.” (Age 27)*
Likely exposure at the clinic	*“That is the problem, not much has changed in terms of taking my pills, the problem is when I need to collect it. It is difficult to leave the house in the morning because you know that you are going to meet up with people with different diseases, you do not know those people’s status, maybe one of them has corona and you will come back with it or maybe you have it and you will go and infect them, you see?” (Age 24)*
Likely exposure in other public spaces	*“Since I am working, I use a taxi all the time, there is no social distancing in the taxi and when I get here at work…I work with customers like everyday, different customers come. I am not safe at all.” (Age 30)*
Likely exposure at work	*“It’s the condition I work under, it is very risky because I work at a store. And when you work at a store some people do not want to wear masks and some don’t even want to sanitize and when a person speaks to you, they come to close to you.” (Age 33)*
Living with family members who do not take COVID-19 seriously	*“There are some who go out when there is no need, go to the neighbours… So, those people can go out and come back with COVID at home…Those people are a problem, no matter hard you shout at them. There is also an aunt who like to galivant, she is old, but she likes going to people’s houses.” (Age 30)*
**MENTAL HEALTH**	
***General decreases in mood***	*“It has affected me a lot because I was not able to cope…I am short tempered, I just cry, I used to think if I were at work, I would not be needing anything. I would just cry until my children would see. You see when you cry in front of your children, the children do not be okay. I would try.” (Age 30)*
***Increased anger due to: Others denying the existence of COVID-19***	*“I wasn’t affected, I know what to do so that I stay safe. I just get angry when I come across a person who says there is no Covid-19 because I can see that its real. There people that say there is no Covid-19, and we are being played with. I wish they would have enough knowledge.” (Age 33)*
*** Increased anxiety due to:***	
Fear of contracting COVID-19	*“In terms of health, I have a fear. I am okay but I am always afraid, once I get a headache or anything or if I cough I would think I have it, I am afraid, is it possible that I have it, so I am always scared if these are the symptoms.” (Age 33)*
Uncertainty about the future	*“You think about how things are going be at the end, will it go back to normal or is it going to be worse when things are finished. I do not know; we wonder what is going to happen. Do you see that?” (7138)*
Financial stressors (e.g., job loss, barriers to finding work)	*“Life is stagnant because you cannot even go out to look for a job, we lost our jobs because of corona. You cannot go to work. Even if you want to go out and try, who is going to employ you now because there are no jobs.” (Age 31)*
Worries about others coping with the impacts of COVID-19 (especially children)	*“Even with the children, there are children who go to school, the children were traumatized, they don’t know what is happening.” (Age 24)*
Unable to continue education	*“Yes, it has affected me because I ended up not being able to complete my studies, I was telling myself that I almost done… But I was unable to…it really caused it…It has affected me…you see the most important thing is my education. It has affected me so much with regards to that.” (Age 28)*
Loneliness/isolation	*“That is the first thing that comes to mind when one is thinking…even if there is something that you want to do…. corona, everything, this corona is in the forefront. So, with regards to my mental state, I would say this is no longer life, it is like we are locked, like we are living in a container. There are things that you can do and there are things that you cannot do.” (Age 25)*
**RELATIONSHIP WITH PARTNER/FATHER OF THE BABY**	
*** Negative impacts***	
Increased physical distance	*“It affected it a lot because I cannot see him for five months. Where he was he could not travel, this was so it is hard… I can feel that he is giving up on us, sometimes he get angry because he cannot see us” (Age 23)*
Poor communication	*“We currently do not get along. We are always fighting over small things, he is a very possessive person, that is why I can say. Like he does not want me to chill with my friends, he calls me, or we will fight about the baby’s food you see, the baby does not have anything to eat. The baby does not have napkins, things like that and he would just beat around the bush.” (Age 23)*
Decreased sexual intimacy	*“Oh no…(laughing) it has affected it because we need to do as per social distance. We are also distancing. We are now like brother and sister, you see.” (Age 39)*
Multiple partnerships/infidelity	*“We are apart from each other, we are not always together since things are like this and he also has a partner in the village, so he likes going home. I am not always around most of them time. Even two months pass by… It makes me feel bad, but I let it pass, I am just focusing on raising my children…If I stress about a man, I might end up in hospital with BP, can you imagine.” (Age 39)*
Financial strain	*“I could say that it gave me a hard time because it led to me fighting with the father of my child because of his job being bad it was that and if it carries on it will not be good because he is struggling to support the children. That’s the negative effect I see.” (Age 33)*
Increased alcohol use	*“I do talk to him but the influence of friends, because when I am with him, we are able to sit without alcohol and he does not even think about it but if I am not close to him for a week, things will get worse.” (Age 25)*
Increased verbal abuse	*“Ah he has never hit me before but sometimes he uses certain words, but I quickly control him because I know he can sometimes speak anyhow you see?… since the COVID-19 outbreak he had trust issues and thought since I am not with him I am with someone else which wasn’t true.” (Age 32)*
*** Positive impacts***	
Increased sexual intimacy	*“But there is no problem between us… Being away from each other is a good thing because it creates and grows the love. We don’t see each other often.” (Age 40)*
Increased quality time	*“It strengthens [the relationship] because we get time to know each other. Spending time with a person without going anywhere, to see what is going on with him, you see, where is he, you see.” (Age 24)*
Improved communication	*“The good that has happened is that we make plans and we sit down and talk about the future… That happens now, but before we did not care about the future, one has to do this and this but now we are able to talk about that.” (Age 21)*
HIV disclosure	*“It helped us, because I managed to tell him about my status and he accepted.” (Age 23)*
Increased help with childcare	*“He focuses on his baby; I don’t want to lie. He supports his baby and makes sure that he covers the baby’s needs.” (Age 39)*
**MOTHERHOOD AND CARING FOR NEW BABY**	
***Lack of social support and/or childcare***	*“Oh, when COVID-19 started and the lock down, I was going to be on maternity leave, I was about to stop working anyway. And then I when the lock down started, I came back and stayed in the farm and I was unable to go back to work… I was working with food. So, it took time for them to open, when they opened, I was not able to find someone who is going to take care of the baby and I ended up not able to go back.” (Age 22)*
*** No or very limited financial support***	*“You see that is something I see is causing stress because the children wake up in the morning and you don’t even have money for bread and the children say they are hungry and you don’t even know where to start and end where that is what affects us the most, the need for food.” (Age 30)*
***Fear that baby/other children will get COVID-19***	*“I am constantly thinking about what to do since my child is not getting immunizations…I am always thinking, I don’t know what to do… So that I can get everything so that I can protect my baby. As I am working there’s a lot of us where I am working. Maybe I will end up getting this virus, maybe I can end up infecting the baby. You see that really hurts me in my heart.” (Age 29)*
***Challenges accessing immunization services for baby due to COVID-19 closures***	*“They kept turning us back and turning us back and they said they are not doing any immunizations. Like, since my child is six months I have gone there. When I arrived, they asked why I haven’t come in to bring the baby, so I went back to the farm because I had shingles and I could not get assistance. Yes, I left because I was not assisted at the clinic, so I ended up going to the farm. When I went to immunize the baby, I told them that they turned me back when Covid-19 started, so I was not advised accordingly from the clinic.” (Age 33)*
***Concerns about breastfeeding while living with HIV in the context of COVID-19***	*“What is important right now and something that I am always cautious about is that I am taking my ARVs accordingly and because the baby is breastfeeding, I always make sure that I am doing it correctly. As well as I how I feed myself. That is what I need to ensure as well as to take care of my family.” (Age 40)*
***Misinformation about newborn health and COVID-19***	*“I would say the COVID-19 had a negative impact in my life because have you heard….do you remember when the COVID-19 arrived, it was everywhere on the social media, they were saying we should not immunise the babies, they are going to kill them, you see. There are those things that spread which are not true. You do not know whether you should take your baby to the clinic or not.” (Age 22)*

#### Engagement in HIV care

The impacts of the COVID-19 pandemic and efforts to manage the spread of the virus in SA impacted engagement in HIV care primarily through reduced access to ART. Often, participants described missing ART doses, either for brief periods or for month-long stretches, because they left the city, went back to rural areas with their families, and did not have access to their typical clinic, where their records are kept. Providers sometimes refused to provide medication if participants did not have their “books” (i.e., their records, which document current medications and doses) with them. Some participants resorted to sharing medications within social networks to help each other. Other challenges to picking up ART were specific to clinic closures, reduced public transportation, and lack of funds for transportation.

#### Physical health

Some participants reported contracting COVID-19 and/or described heightened risk for acquiring COVID-19, which had either direct or potential downstream effects on engagement in care. Most participants were acutely aware that their HIV-positive status increased their risk for negative COVID-19 outcomes. Participants were concerned about possible COVID-19 exposure at work, in public spaces where social distancing was not feasible, and at home, particularly among family members who did not take COVID-19-related precautions seriously. Moreover, participants expressed fear and uncertainty around possible COVID-19 exposure at the clinic, especially when collecting their medications.

#### Mental health

Themes specific to general decreases in mood, increased anger, and increased anxiety also emerged from the data. Participants expressed the high toll that the pandemic and associated lockdowns have taken on their emotions, increasing the intensity of expressed emotions and, for example, complicating efforts to hide sadness, anger, or fear from their children. Many factors were cited as catalysts for increased anxiety, including fear of contracting COVID-19, concerns that others (especially children) might cope poorly with COVID-19, and lack of financial resources, as many participants lost their jobs. Women also expressed multiple levels of uncertainty about the future, including potential vulnerabilities due to the interaction of COVID-19 and HIV, impacts on educational attainment, and the impacts of continued isolation.

#### Relationship with partner/father of the baby

When asked about the effects of the pandemic on partnerships and/or the relationship with the father of the baby, participants described both negative and positive impacts, with descriptions that often conflicted. The majority of the sample noted decreased, rather than increased, interaction with their intimate partners, as many were restricted from traveling and had partners who were working in other cities or outside of the country. Some women described the ways in which decreased in-person interaction led to concerns that their partners would leave them, forget about their children, and fail to provide financial support, which was a major concern. Increased distance was also linked to decreased sexual intimacy with partners, an increase in suspected relationships between male partners and other women, verbal abuse, and increased alcohol use among male partners. Other participants, however, noted that increased distance improved their relationships. A minority of women experienced lockdown with their partners; some of these participants reported improved communication and joint planning for the future, disclosure of HIV status, and increased assistance with childcare.

#### Motherhood and caring for the new baby

Themes associated with childcare challenges were characterized by lack of support, fears that infants would acquire COVID-19, and concerns about breastfeeding when ART access is limited. Participants described consistent lack of support throughout the different lockdown periods, both specific to childcare, which prohibited work, and to finances, which made it almost impossible to meet infants’ needs. As women were unable to secure immunizations for their infants, they feared that their babies would be more susceptible to COVID-19. Similarly, with decreased consistency in ART use, again, due to decreased access, participants worried about HIV transmission via breastfeeding but did not report specific changes in breastfeeding behavior. Misinformation on social media about infant immunization in the early phases of the pandemic made it difficult for some participants to assess whether taking their babies to the clinic posed more or less risk than avoiding immunizations all together.

#### Coping

When asked how they coped with these COVID-19-related challenges, participants elucidated several strategies. See Table 4 for descriptions of each of the coping strategies that participants employed.

**Table 4:** Coping with the impacts of COVID-19

Domains & associated themes	Illustrative quotes
**SPECIFIC COPING STRATEGIES**	
Social support	*“[My current partner] fills that gap that the father of my baby abandoned me, you see. So, I no longer have stress about not having the father of my baby… I mean I am not expecting him to give me money for the baby, but the stress of not having no one, he closes that gap.” (Age 33)*
Acceptance	*“We have to just accept it; you cannot cope with everything. I cannot say I am satisfied because, as a mom, I am not working. I am not satisfied because we are not in a stage where I want us to be in. I am grateful that [my baby] is alive, she is able to eat, she does not go to bed without food. That is what I can say.” (Age 31)*
Spirituality, prayer, or religion	*“For now, I don’t know what is it that can be done, what could make it better. Nothing beats prayer, to pray for myself, that makes it better until this lockdown passes.” (Age 29)*
Adherence to COVID-19 prevention protocols	*“I do not go anywhere when there is no need, I make sure that I stay at home. When I go out, I have my mask. I take my baby to the clinic or when I go to collect my pills. I only go out when there is a need.” (Age 22)*
Distraction or engagement in activities	*“What I used to do to keep myself busy [is] I opened a space for a garden and planted vegetables so that when I want something, I am able to get it, because I am not able to get anything because of this corona.” (Age 28)*

Participants described their use of specific coping strategies to decrease the distress associated with reduced engagement in HIV care, negative mood, evolving relationships with their partners, and challenges meeting their infants’ needs. These strategies were also used to cope with financial limitations and associated distress around meeting their family’s basic needs—especially the baby’s needs. Some women intentionally sought social support from family, friends, and new partners, whereas others relied heavily on spirituality and prayer. Many participants described taking active steps toward acceptance, recognizing that they had little to no control over the course of the pandemic and expressing gratitude for what they do have, rather than focusing on that which they have lost. Some found solace in adherence to COVID-19-related protocols, and still other participants coped via adaptive distractions, such as gardening.

## Discussion

This analysis of multi-method data collected from postpartum WWH who were enrolled in a longitudinal cohort study revealed that about 20% of the cohort faced COVID-19-related challenges accessing HIV care, medications, or infant-related services. Brief qualitative interviews were conducted with the participants who endorsed those challenges to more thoroughly understand their experiences. With respect to direct impacts on care engagement, participants described challenges with ART use, including missed doses and shared pills with friends, as well as numerous logistical barriers to accessing their medications, often because they moved away from the vicinity of their home clinic and were refused medications from other clinics. Participants also described COVID-19-related impacts on factors that may indirectly influence care engagement, including physical and mental health, intimate relationships or partnerships, and new parenthood. With awareness of the association between HIV and severe COVID-19-related outcomes, many participants were concerned about their health and possible COVID-19 exposure, especially at the clinic. Negative effects on mental health included increased sadness, anger, and fear, with intersecting uncertainties—unknown interactions between COVID-19 and HIV, unknown impacts of continued isolation, unknown employment or financial prospects—leading to a lack of hope for the future. Effects on relationships with partners were mixed, with some participants reporting strong negative consequences of decreased interactions with their partners (e.g., feared or confirmed infidelity, lack of financial support for the infant, verbal abuse) and others expressing positive experiences when cohabitating with partners during lockdowns (e.g., increased communication, increased intimacy, HIV status disclosure). Finally, effects on parenting the new infant mostly centered on lack of financial resources, which limited participants’ ability to provide food and clothing, decreased access to immunizations due to COVID-19-related closures or restrictions on travel, and concerns about transmitting COVID-19 via breastmilk. Finally, when asked how they coped with these COVID-19-related challenges, they described highly adaptive strategies, including seeking social support, moving toward acceptance, and spirituality.

Though 20% is a sizeable proportion of participants who experienced barriers to care engagement, we suspect that it is likely an underestimation of the level of disruption, especially during the initial lockdown periods. Within the first few months of the pandemic, researchers and clinicians based in sub-Saharan Africa and other regions with high HIV prevalence rates signaled the alarm, drawing attention to the potential for severe disruptions at each phase of the HIV care continuum—from HIV testing to ART access and interrupted ART supply to attrition from care and HIV-related deaths [32–34]. Several commentaries described the ways in which the pandemic might exacerbate structural inequities and HIV disease burden, particularly among women, who are more likely to be living with HIV compared to men and who typically serve as frontline workers in low- and middle-income countries [13, 35]. By the time we collected our data (especially the data that were collected in the latter part of Wave 1 and Wave 2), some participants may have been able to effectively navigate the initial barriers, and their retention in the parent study throughout the most intense phases of the pandemic may indicate higher than average levels of resilience. Even so, the engagement in care findings validate early concerns and recent findings on the impacts of the pandemic and associated public health efforts to manage disease transmission on the HIV treatment cascade. Indeed, reductions in HIV testing at first antenatal visit and reductions in HIV treatment access during pregnancy were documented across 17 countries and 15 countries, respectively [36]. Recent quantitative data from 65 primary care clinics in KwaZulu-Natal, where the data for the current analysis were collected, provides a broad-scale view of the impacts of the 2020 national lockdowns on HIV testing and treatment. In the first week of lockdown (March 30, 2020 to April 5, 2020), there was an estimated 47.6% decrease in HIV testing and a 46.2% decrease in ART initiation [37], suggesting that the bulk of the impact may have been felt in the earlier phases of the HIV treatment cascade. In the large primary care sample, ART collection visits decreased only slightly and missed ART collection visits increased for just a short time [37], suggesting that ART provision was largely maintained during this specific time period. This may explain why more women in our sample did not endorse challenges collecting their ART, though we did find that ART access was compromised for some. It remains unclear, however, if these patterns changed over subsequent periods, and the degree to which specific subpopulations that face additional barriers to retention in care under normal circumstances (including postpartum WWH [8]) were more severely impacted than others was not examined.

Our findings also reinforce the importance of attending to and providing resources to address the mental health of women with HIV during public health crises. General decreases in mood may have downstream effects on engagement in HIV care, with potential for heightened risk of dropout among postpartum WWH, a population that faced significant mental health challenges pre-COVID-19. For example, in a review and meta-analysis published in early 2020, the pooled prevalence of postpartum depression in Africa was 16.8% [38], with pre-pandemic rates specific to SA hovering between 35 and 47% [39]. Depression is a known barrier to engagement in HIV care [40], and depression during the postpartum period—both related and unrelated to COVID-19—has strong negative implications for decreased ART adherence and potential perinatal transmission [41]. In addition to decreased mood, women in our sample understandably also expressed specific worries and concerns about potential HIV/COVID-19 co-infection, finances, and their ability to finish their education as well as plan for their child’s future. These worries and more general anxiety, though normative in the early phases of the pandemic, may eventually contribute to patterns of behavioral avoidance, leading to further reduced access to HIV-related care. In addition, the combined effects of intimate partner violence and sexual trauma, which are common among WWH in SA [42, 43], as well as rekindled memories of apartheid-era restrictions may also compromise care engagement [13]. Though we did inquire about physical violence from a partner during the lockdown period, we did not explore the degree to which other forms of violence, pre-COVID-19 traumas, or posttraumatic stress may have contributed to or exacerbated difficulties accessing care. Nonetheless, it is evident that the pandemic contributed to poor mental health among postpartum WWH.

Although some participants highlighted positive effects of the COVID-19 lockdowns on their current romantic relationships, most women emphasized negative consequences, financial stressors resulting in part from greater physical distance from partners, and a strong need for additional childcare support. For a minority of participants, increased quality time with partners facilitated improved communication, increased intimacy, and HIV status disclosure. These benefits have not been widely reported in the existing literature, which has primarily focused on the ways in which the pandemic has jeopardized relationship quality and stability [44, 45], nor have they been discussed among sub-populations at heightened risk for negative COVID-19 outcomes. In some cases, COVID-19 may have offered couples the opportunity to join together against an external threat [44], especially if partners were locked down together. But, for the most part, women in this sample described the negative effects of decreased interaction (e.g., partners initiating sexual relationships with other women, verbal abuse, increased alcohol use) as the majority of participants were locked down separately from their partners. Similarly, in a Kenyan sample of adolescent girls and young women who had romantic partners during the pandemic, reduced time with partners was the strongest predictor of decreased relationship quality [46]. For many participants, decreased interaction with partners also translated into lack of shared childcare responsibilities and decreased financial support for the infant, exacerbating existing gender inequalities, particularly in the unpaid (care) economy. Not only did women in SA experience two thirds of the net job losses between February and April 2020, they also took on a disproportionate share of additional childcare following school closures [47].

Other notable negative effects of the pandemic on parenting during the COVID-19 lockdowns were significant confusion around and reduced access to infant immunization, concerns about missed ART doses in the context of breastfeeding, and fears about infecting the baby as well as other children. Finding balance between guarding against the spread of COVID-19 and controlling well known preventable diseases has proved challenging, particularly in low resource settings, with recent modeling predicting that not maintaining routine infant immunization will lead to more deaths than deaths related to COVID-19 exposures at vaccination clinics [48]. In a survey of members of the Immunizing Pregnant Women and Infants Network (IMPRINT), over 75% of whom were based in low- and middle-income countries, 50% reported broad challenges accessing immunizations, including logistical barriers, provider issues, and not attending appointments due to COVID-19 fear [49]. Although participants did not describe changes to their breastfeeding behaviors, there were clear concerns that limited access to ART in the context of the pandemic would render their breastmilk unsafe, and with decreased financial resources to purchase formula, for example, the health of their infants could be at risk.

Importantly, the range of coping strategies that participants described demonstrate incredible sources of strength and resilience in spite of limited access to infant care services as well as reduced access to HIV-related care, physical and mental health concerns, and changing relationship dynamics. A combination of social support, acceptance, strength through prayer, steadfast adherence to COVID-19 guidelines, and intentional distraction with other activities (e.g., cooking, gardening, cleaning) enabled participants to navigate through some of the darker periods of the lockdowns. Similar strategies were used by survivors of the 2014–2016 Ebola outbreak in West Africa, which primarily impacted Guinea, Liberia, and Sierra Leone [50]. During this period, individuals affected by Ebola maintained active involvement in community prevention efforts, participated in prayer and bible study, and sought social support from both family members and non-governmental organizations [51]. Among survivors of the SARS epidemic in Hong Kong, socialization through activities like Tai Chi helped restore a sense of meaning to their lives [52]. Participants who identify what is important to them and what makes them feel good, even when confronted by a situation that they cannot control, may enable them to pursue meaningful goals and activities under extremely trying circumstances [53, 54].

Several limitations of the current analyses should be noted. Small sample sizes are typical of qualitative work, but the size of our sample (specifically the small size of our COVID-19 wave 2 sample) limited our ability to separate the data by lockdown or by phase of the COVID-19 pandemic. Therefore, we could not draw conclusions about engagement in HIV care and contributing psychosocial challenges that may have been unique to specific time points over the ongoing pandemic’s duration. Similarly, based on the timing of their enrollment in the parent study, participants completed the COVID-19 assessment and corresponding interview at different points in their postpartum experiences (6, 12, 18, and 24 months), with too few interviews at each timepoint to explore relationships between early vs. late postpartum and COVID-19-related barriers to care. We had fewer participants complete assessments at the 24-month follow-up period (*n* = 5) relative to the other follow-up assessments (18, 13, and 17, respectively), for example, because we had hopes of conducting this final assessment in-person. The quantitative assessment was not psychometrically validated; the questionnaires that we adapted (i.e., items modified from the N2 COVID-19 Check-In Survey Items and the Adolescent Trials Network COVID Questionnaire Draft*)* were working documents with undefined response items that were developed by researchers and posted online during the early phases of the pandemic for others to use in their studies. Even though the quantitative assessment was not the focus of this sub-study, any conclusions drawn from non-validated tools should be interpreted with some degree of caution. Importantly, participants who were not included in this qualitative sub-study either endorsed none of the four engagement in care challenges (making or keeping their HIV care appointments, procuring their HIV medications, procuring contraception, or accessing immunization services for their infants) or did not complete the parent study assessment to which the COVID-19 questions were added. It is possible that participants had challenges with other aspects of HIV- or infant-related care or follow-up that were not included on the list and therefore were not documented. It is also possible that participants who did not attend their parent study assessment may have had different or worse experiences than those who completed the assessments. Therefore, even though we selectively identified participants who did report difficulties remaining in care or accessing treatment, there may have been a selection bias toward a more resilient sample, such that postpartum WWH who were lost to follow-up in the parent study may have had worse COVID-19-related outcomes.

In conclusion, a significant portion of postpartum WWH have faced challenges making or keeping their HIV care appointments, procuring their HIV medications and/or contraception, and accessing immunization services for their infants during the early waves of the COVID-19 pandemic in SA. COVID-19-related effects on physical and mental health, relationship with partners, and parenting/childcare are both important to address in their own right and may have critical implications for both retention in HIV care and prevention of perinatal transmission. It is also important to highlight the degree to which the pandemic affected the financial wellbeing of this sample, rendering it ever more challenging to meet basic needs. As the pandemic continues, providers who serve this population (who were also under tremendous pressure during this time [55, 56] and are therefore in need of continued support) and public health officials who set HIV care policy should proactively address these concerns at individual and systems-levels to avoid disruption of services, especially essential services for populations already at risk of attrition from HIV care.

## Data Availability

The datasets used and analyzed during the current study are available from the corresponding author on reasonable request.

## Abbreviations

ART: Antiretroviral therapy/ies
COVID-19: Coronavirus disease 2019
HIV: Human immunodeficiency virus
IMPRINT: Immunizing Pregnant Women and Infants Network
IPV: Intimate partner violence
PMTCT: Prevention of mother to child transmission
PWH: People with HIV
RNA: Ribonucleic acid
SA: South Africa
USD: United States dollar
WWH: Women with HIV
ZAR: South African Rand
